# The Prevalence of Lunacy in England and Wales

**Published:** 1902-09-20

**Authors:** 


### THE PREVALENCE OF LUNACY IN ENGLAND AND WALES.

The fifty-sixth report of the Commissioners in Lunacy (1902) which has recently been issued gives, as usual, full details regarding the prevalence and increase of lunacy. The average number of patients resident in all institutions for lunatics, idiot-establishments, and in single charge in 1901 was 86,600, and the total deaths were 8,342. Excluding patients in idiot-establishments the mortality-rate of the year was 9·77 per cent. of the average number of patients resident, being 0·37 per cent. below the rate in 1900. As to the question of the increase in the prevalence of lunacy the returns, of course, include only such cases as are certified. The total of all lunatics notified to the Commissioners has increased from 36,762 on January 1st, 1859, to 110,713 on January 1st, 1902. While the population has increased, the proportion of lunatics to population has increased still more. In 1859 the proportion was one lunatic to 536 persons, and last year the proportion was one lunatic to 301 persons. But this great advance has been almost entirely in the pauper class.

